# Eating Egg-Rich Diets and Modeling the Addition of One Daily Egg Reduced Risk of Nutrient Inadequacy among United States Adolescents with and without Food Insecurity

**DOI:** 10.1016/j.tjnut.2024.09.019

**Published:** 2024-09-21

**Authors:** Analí Morales-Juárez, Alexandra E Cowan-Pyle, Regan L Bailey, Heather A Eicher-Miller

**Affiliations:** 1 Department of Nutrition Science, Purdue University, West Lafayette, IN, United States; 2 Institute for Advancing Health Through Agriculture, Texas A&M University System, College Station, TX, United States

**Keywords:** food security, nutrient intake, eggs, adolescents, NHANES

## Abstract

**Background:**

Adolescents have the poorest dietary intake throughout their lifespan. Food insecurity worsens these nutritional risks. Eggs are a nutrient-dense strategy to increase nutrient quality.

**Objectives:**

*1*) compare usual nutrient intakes, Dietary Reference Intake and protein compliance with recommendations and scores of micronutrient quality; and *2*) analyze how adding 1 egg affects adolescents’ nutrient profiles, by food security status and egg-rich diets.

**Methods:**

Dietary data of United States adolescents in the 2007–2018 National Health and Nutrition Examination Survey were analyzed (14–17 y; *n* = 3633). Egg-rich diet levels were categorized as *1*) no eggs, *2*) eggs as ingredients in dishes, or *3*) primarily egg dishes. Food security status was classified using the United States Household Food Security Survey Module. The National Cancer Institute method was used to estimate usual nutrient intake and nutrient exposure scores [i.e., Food Nutrient Index (FNI) and Total Nutrient Index (TNI)]. Nutrient amounts from 1 medium egg were modeled on existing intakes. Pairwise t-tests determined significant differences.

**Results:**

Over 60% of adolescents risked inadequate intake of calcium, choline, magnesium, vitamin D, and vitamin E regardless of food security status. Food-secure adolescents consuming primarily egg dishes had higher mean usual intakes of lutein + zeaxanthin (1544.1 *μ*g), choline (408.4 mg), vitamin B2 (2.3 mg), selenium (128.6 *μ*g), vitamin D (6 *μ*g), docosahexaenoic acid (70 mg), and protein (89.1 g) than other groups (*P* < 0.0002). Those who were food secure and consuming eggs as ingredients in dishes demonstrated higher nutrient adequacy for magnesium (scored ∼66 out of 100), potassium (scored 81), and total scores (scored 72 and 69, respectively) for the TNI and FNI; and folate only (scored 92) for the TNI, than those who were food insecure and not consuming eggs (*P* < 0.0002). Adding 1 egg increased choline and vitamin D usual intakes for some groups and nutrient index scores for all groups (*P* < 0.0005).

**Conclusions:**

Adolescents are at substantial nutritional risk that was exacerbated by food insecurity and less egg consumption.

## Introduction

Food insecurity is a major United States public health concern; 17.3% of households with children and adolescents (0–17 y; 6.4 million Americans in total) in 2022 [1] experienced food insecurity, a situation that occurs when a household does not have the resources to keep adequate food for all members [1]. The Dietary Guidelines for Americans (DGA), which provides science-based recommendations on food and beverage choices to support health, lower risk of chronic diseases, and fulfill nutrient requirements, highlights that adolescence is the life stage of lowest diet quality and nutrient inadequacy for multiple food components and nutrients [2]. This is concerning given the rapid growth and development that occurs in these years and the need for adequate nutrition to promote health [2]. For example, the Scientific Report of the 2020 DGA Advisory Committee [3] described that United States adolescents experienced shortfalls of nutrients of public health relevance for protein, iron, folate, vitamin B6, vitamin B12, phosphorus, magnesium, choline, calcium, vitamin D, and potassium [3,4]. Adolescents are also less likely to use a dietary supplement (DS) to achieve nutrient recommendations compared with other life stages [5,6]. Given that the overall quality of the diet is low, the additional nutritional risk presented by food insecurity may magnify these and other nutrient gaps among adolescents. Only a few studies have focused on identifying vulnerable population groups by age and food security status [7,8]. Hence, an objective of the present study was to characterize and compare usual nutrient intakes, the proportion of the population at risk of nutrient inadequacy, and a diet and total diet (nutrients from diet and supplements) nutrient exposure score by food security status among United States adolescents.

Practical and accessible nutrient-dense foods that provide nutrients that are commonly under-consumed are important to consider for potential inclusion in dietary interventions, specifically those that aim to improve Dietary Reference Intake adherence among United States adolescents. Another objective of this work was to examine the role of eggs in adolescent nutrient intake and adherence to nutrient adequacy markers, given that eggs are a rich source of many of the nutrients of concern in this life stage. Eggs represent a low-cost, nutrient-dense food that is commonly consumed as a part of the United States dietary pattern [9] and contribute to protein intake as well as intakes of numerous micronutrients (e.g., choline) [10]. Eggs, as part of the protein group, are included in meal plans such as the Thrifty Food Plan (TFP), which outlines nutrient-dense foods and beverages while also considering the costs and affordability of supporting a healthy diet [11].

Although dishes primarily comprising eggs (e.g., hard-boiled eggs) are considered protein foods, eggs are commonly included as ingredients in other types of dishes (e.g., burritos), as a component of foods in the grain group (e.g., bread) and a lesser extent some snack foods [12]. This varied distribution of eggs across several food groups presents challenges in terms of understanding their nutrient intake contributions. Prior research has shown that modeling the addition of 1 egg/day to the diets of United States children (2–18 y) provides meaningful contributions to usual intakes of choline, lutein, and zeaxanthin respective to the current United States dietary patterns [13], and increases intakes of selenium, vitamin B2, and vitamin D among those 1–18 y [14]. Nevertheless, eggs provide several additional nutrients that have yet to be evaluated in this context, and few studies, if any, have modeled the impact of the addition of 1 egg on usual nutrient intakes among United States adolescents.

Therefore, the objectives of the present analysis were to *1*) estimate and compare usual nutrient intakes, the proportion meeting Dietary Reference Intakes for micronutrients and for protein, the proportion meeting the DGA and TFP recommendations, and nutrient exposure scores for diet and diet and supplements; and *2*) model the impact of adding 1 egg on these nutrient profiles, among United States adolescents (14–17 y) who participated in the 2007–2018 National Health and Nutrition Examination Survey (NHANES) by food security status (food secure compared with food insecure) and degree of egg-rich diets (nonegg consumers, consumers of eggs as ingredients in dishes, and consumers of primarily egg dishes).

## Methods

### Participants and data collection

The NHANES is a continuous, cross-sectional series of surveys of the resident, civilian, and noninstitutionalized United States population that can be used to provide nationally representative estimates to monitor the health and nutritional status of the nation [15]. Data collection in the NHANES consists of an in-home interview, a health examination in the mobile examination center (MEC), and a follow-up telephone interview. All participants and/or proxies consented to participate in the survey, and all methodological procedures were approved by the National Center for Health Statistics institutional review board [16]. Six survey cycles prior to the COVID-19 pandemic, which was known to cause changes in dietary intakes, were combined for this analysis (i.e., 2007–2008, 2009–2010, 2011–2012, 2013–2014, 2015–2016, 2017–2018) to increase the reliability and stability of statistical estimates across subgroups [17]. This study encompasses 2 analytic samples. The first one was used to estimate the mean usual nutrient intake and the percentage of adolescents not meeting the nutrient recommendations and included adolescents aged 14–17 y with complete food security data (i.e., household child food security) and ≥1 reliable 24-h dietary recall (*n* = 3633). The second sample was employed to estimate the Total Nutrient Index (TNI) and Food Nutrient Index (FNI) scores and consisted of adolescents aged 14–17 y with complete information on DSs [i.e., DS and Prescription Medicine Questionnaire (DSMQ) and with ≥1 24-h dietary recall (*n* = 1822) (Supplemental Figure 1).

### Sociodemographic variables

Demographic and socioeconomic data were collected through an in-home interview using the interviewer-administered computer-assisted personal interview system [15]. A proxy was reported for those aged ≤15 y old, whereas those ≥16 y answered the questions for themselves. Self-reported race/Hispanic origin was categorized as Mexican American, other Hispanic, non-Hispanic White, non-Hispanic Black, and “other” racial/ethnic categories. The family poverty-to-income ratio (PIR) represents the ratio of the annual family income to the poverty guideline established by the Department of Health and Human Services [18]. Four PIR categories were constructed: <1.00, <1.30, <1.85, and ≥1.85. A PIR of ≤1.30 indicates potential eligibility for the Supplemental Nutrition Assistance Program (SNAP), a program offering cash benefits for foods to low-income households to reduce food insecurity [19]. The household’s current SNAP participation status variable was used to identify program participants. The National School Lunch Program and the National School Breakfast Program (NSLP/BP) provide meals at reduced cost to school children in households with a PIR of 1.31–1.85 and free meals to those in households with a PIR ≤1.30. Adolescents aged 14–17 y who were enrolled in the NSLP/BP, receiving either reduced-cost or complementary meals, were recognized as NSLP/BP beneficiaries [19]. BMI (in kg/m^2^) was calculated via height (m^2^) and weight (kg) measurements collected during the physical health examination in the MEC. Obesity was defined as a BMI greater than or equal to the age and sex-specific 95th percentile of the 2000 Centers for Disease Control and Prevention Growth Charts [20].

### Food security measurement

Food security was measured using the United States Household Food Security Survey Module during the in-home interview [21]. An adult head-of-household responded to the 10 items for the entire household, and an adult proxy answered the additional 8 items with regard to the items specific to children in the household. When the child was ≥16 y, they could answer the 8 questions specific to children in the household themselves. Each question referenced the past 12-mo period. The ranges of experience of the entire household and children in the household were classified into 1 of 4 categories of food security (i.e., high, marginal, low, and very low) [22]. High and marginal food security categories were combined to classify food security, and low and very low food security categories were combined to classify food insecurity. The food security of household children was used in this study as a more direct and sensitive measure, compared with household food security, that would be better aligned to children’s experience and dietary intake [[23], [24], [25]].

### Diet and DS assessment

Dietary data on foods, beverages, and DS is collected via ≤2 24-h dietary recalls [26]; in NHANES 2007–2018, the first 24-h recall was interviewer-administered in person in the MEC, whereas the second recall was completed via telephone ∼3–10 d later. Both recalls were collected using the validated, USDA Automated Multiple-Pass Method [26,27], and participants were asked to report all foods, beverages, and DS consumed from midnight to midnight the previous day. For the 24-h dietary recalls, the participants aged ≥12 in this study could answer for themselves. DS intake over the previous 30 d is also assessed via the DSMQ. A proxy provided information for children aged ≤15, whereas those aged ≥16 answered the questions on their own. DS users were identified by whether participants indicated “yes” to consuming any products containing the specific nutrient of interest on the DSMQ [28]. The nutrient intakes of all reported foods and beverages for each participant were determined using the USDA’s Food and Nutrient Database for Dietary Studies (FNDDS) for data from 2007–2008 (version 4.1), 2009–2010 (version 5.0), 2011–2012, 2013–2014, 2015–2016, and 2017–2018 [29]. Nutrient values for DSs were calculated using the NHANES DS database [30,31].

### Egg-rich diets

Dietary data from the 24-h recall, day 1, was used to categorize mean participant level of egg-rich diet as not including eggs, including eggs as ingredients in dishes, and including primarily egg dishes. The eggs as ingredients category included foods where varying amounts of eggs were included but where the egg was not the primary ingredient in any food consumed (i.e., egg burritos, egg sandwiches, pastries, etc.). All foods, including the food description “low fat,” “cholesterol free,” “reduced fat,” and “fat-free,” were excluded from the analysis given that it could not be confirmed if eggs were removed or included in those dishes. Primarily egg dishes were classified when participants reported eating eggs defined by FNDDS food category 2502, “eggs and omelets.” However, within the 2502 food category, some individual foods were excluded as follows: “egg substitute, NS (not specified) as to powdered, frozen, or liquid” and other poultry eggs (e.g., “duck egg,” “goose egg,” and “quail egg”). If an adolescent reported consuming a food where eggs were an ingredient and a primarily egg dish, they were classified in the primarily egg dishes category only. If an adolescent reported consuming >1 dish where eggs were an ingredient, they were still included in the category of consuming eggs as ingredients.

### Modeling the addition of 1 egg

Egg-rich diets were enriched with the addition of all respective nutrients comprising 1 medium egg 50 g/d, “egg, whole, boiled or poached” food category 31103010, from the most current version of the FNDDS 2019–2020 [32]. This type of egg represented the most frequently reported type of egg consumed among adolescents in the sample at 7%, a proportion tied with 7% also reporting intake of “egg omelet or scrambled egg, made without fat,” but the boiled or poached egg was chosen due to the basic preparation without the addition of other dietary components (e.g., seasonings, etc.).

### Estimating usual intake and the proportion meeting adequacy markers

To estimate means and distributions of usual intake at the group level from a limited number of self-reported 24-h recalls per individual, statistical modeling techniques were applied to mitigate the influence of random measurement error (e.g., day-to-day variation). The National Cancer Institute (NCI) method [33] approximated usual intakes for nutrients of public health relevance and those that low-income populations are at high risk of inadequacy, including calcium, choline, DHA, folate, iron, lutein + zeaxanthin, magnesium, potassium, protein, selenium, and vitamin A, vitamin B1, vitamin B2, vitamin B3, vitamin B6, vitamin B12, vitamin C, vitamin D, and vitamin E, and zinc [[34], [35], [36], [37], [38], [39], [40]]. Covariates included the recall day (i.e., weekday compared with weekend) and sequence of the recall (i.e., first compared with second). The other variables for characteristics that were significantly differentially distributed among the groups were evaluated and showed correlation with each other. So, in order to avoid multicollinearity and to preserve the best estimation of the mean usual intake for these United States population subgroups, adjustments for these sociodemographic characteristics were not made to estimates of the usual intake or comparisons among groups. The NCI method produces means and percentiles of the usual intake and proportions of the group with intakes below the Estimated Average Requirement (EAR) or above the Adequate Intake (AI). The AI is the average daily nutrient intake for a healthy population that is assumed to be adequate for the population’s needs, and AI is used when the evidence is not sufficient to establish an EAR [41]. Fay’s modified balanced repeated replication technique was performed to compute SEs [42,43]. The percentage of intakes below the EAR when the EAR is established, and the percentage above the AI was used as an indicator of the percentage at risk of inadequate intake using the cut-point method [41].

The proportion of adolescents meeting the DGA and the TFP protein recommendations was estimated in a similar manner. For the DGA, adolescent females’ energy needs are estimated at 1800–2400 kcal/day, and for males, ∼2000–3200 kcal/day [2]. Considering the sedentary behavior of adolescents [44, 45] and that adolescent girls have lower protein intake than boys [2], a lower kilocalorie level was chosen to conservatively approach kilocalorie and protein needs. The DGA utilizes the Acceptable Macronutrient Distribution Ranges for protein, which is 10–30% of total calories, with a minimum of 46 g for adolescent girls in 1800-kcal/day and 52 g for boys in 2200-kcal/day. Hence, 46 g for girls and 52 g for boys were used as cut points to estimate the percentage of adolescents meeting the DGA protein recommendation in this study. The TFP, upon which SNAP is based, embodies a balanced diet on a limited food budget for a household with 2 adults, a male and female aged 20–50 y, and 2 children aged 6–8 and 9–11 y [11]. The TFP recommends 55 g of protein for girls and 75 g for boys, based on 2189 and 2985 kcal menus, respectively. Thus, 55 g for girls and 75 g for boys were applied as thresholds to calculate the proportion of adolescents who met the TFP protein recommendations [11].

### Total Nutrient Intake and Food Nutrient Intake estimation

In addition to these nutrient profile indicators, the nutrient quality from diet and from diet and supplements was examined. The TNI and FNI are nutrient-based scoring metrics designed to assess usual intakes of under-consumed micronutrients among the United States population relative to the Recommended Dietary Allowance (RDA) or AI and are identical in terms of index construction, with the main difference being that the TNI assesses total usual intakes from foods, beverages, and DS, where the FNI evaluate usual intakes from foods and beverages alone [28,46]. Thus, total usual micronutrient intakes from all sources can be contrasted with those from dietary sources when utilizing both scores [28,46]. For the TNI and FNI, 8 micronutrients were prioritized for inclusion: calcium, magnesium, potassium, zinc, choline, folate, vitamin C, and vitamin D, based on the high percentage of underconsumption for adolescents in the different food security and egg-rich diet groups. The TNI and FNI have previously demonstrated relative validity; scores differentiate groups with known differences in nutrient intake and correlate well with biomarkers of nutritional status [28,46]. For the TNI and FNI score estimation, the mean usual nutrient intakes were evaluated as a percentage (≤100%) of the RDA or AI (values for adolescents 14–17 y), with higher scores indicating better adherence to the RDA or AI. Each nutrient received a mean TNI and FNI score, then all of the mean nutrient scores were averaged to determine the total scores where all micronutrients were equally weighted [28,46].

### Statistical analysis

The proportions of the sociodemographic variables were evaluated using the Rao-Scott F adjusted χ^2^ tests for categorical variables to compare by food security status and the egg-rich diets, with a 2-sided *P* value of <0.05. This step also served to identify potential confounders that were considered in comparison to the usual intakes. Pairwise t-tests were carried out to determine the differences in usual nutrient intake, proportion of adolescents meeting the EAR or AI, and TNI and FNI scores between household child food security status groups and then further for food security by egg-rich diet groups (food-insecure nonegg consumers, food-insecure consuming eggs as ingredients in dishes, food-insecure consuming primarily egg dishes, food-secure nonegg consumers, food-secure consuming eggs as ingredients in dishes, and food-secure consuming primarily egg dishes) and for modeling the addition of 1 egg. A Bonferroni-adjusted *P* value, based on the number of comparison groups across all of the nutrients or nutrient scores analyzed, was applied to account for multiple comparisons. All statistical analyses were performed using SAS 9.4 software (SAS Institute). To ensure that the findings represent the entire population, survey weights were applied along with adjustments regarding the variance estimation and subsetting data to account for the complex sample design of NHANES [47].

## Results

### Food security status

Fourteen percent of United States boys and girls aged 14–17 y were living in a household with food insecurity from 2007 to 2018. Compared with food-secure adolescents, those who experienced food insecurity were more likely to be Mexican American or non-Hispanic Black, living in lower-income households (i.e., PIR <1.00), living in households participating in SNAP, participating in the NSLP/BP, with a BMI classified as obese; and a lower proportion of consumers for primarily egg dishes and eggs as ingredients in dishes. Among all adolescents, 49% were nonegg consumers, 36% consumed eggs as ingredients in dishes, and 15% consumed primarily egg dishes. In the group of adolescents experiencing food insecurity, 51% were nonegg consumers, 32% consumed eggs as ingredients in dishes, and 17% consumed primarily egg dishes. In the food-secure group, 47% were nonegg consumers, 39% consumed eggs as ingredients in dishes, and 14% used primarily egg dishes (Table 1).

**TABLE 1:** Characteristics of United States adolescents aged 14–17 y by household child food security status, National Health and Nutrition Examination Survey 2007–2018^1^

Characteristic	Food insecure			*P* value^2^	Food secure			*P* value^2^
	Non-egg consumers	Eggs as ingredients in dishes	Primarily egg dishes		Non-egg consumers	Eggs as ingredients in dishes	Primarily egg dishes	
	*n* % ± SE	*n* % ± SE	*n* % ± SE		*n* % ± SE	*n* % ± SE	*n* % ± SE	
Total	264	168	88		1504	1141	468	
	51.0 ±2.1	32.4 ± 1.8	16.6 ±1.7		47.0 ±1.1	39.0 ±1.2	14.0 ±0.8	
Sex				0.35				0.68
Boys	128	87	52		777	578	244	
	24. 0±2.3	17. 3± 1.9	9.2 ± 1.4		23. 3± 0.9	19. 1±0.9	7. 1±0.5	
Girls	136	81	36		727	563	224	
	27.0 ±2.4	15.0 ± 1.6	7. 5± 1.3		23.5± 1.0	20.0 ±0.9	7.0 ± 0.6	
Racial/ethnicity				0.85				0.0007
Mexican American	91	46	28		321	218	104	
	14.0 ±1.7	7.1 ± 1.4	4.2 ± 0.8		6.2 ± 0.6	4. 0±0.4	2. 0± 0.2	
Other Hispanic	28	20	11		144	107	61	
	4.3 ±1.0	3.0 ± 0.7	2.0 ± 0.6		3.0 ± 0.3	2.0 ±0.2	1.1 ± 0.1	
Non-Hispanic white	61	42	16		433	392	114	
	20.3 ±2.4	13.2 ± 2.3	5.3 ± 1.2		27.0 ± 1.2	25.0 ±1.4	7.2 ± 0.7	
Non-Hispanic black	67	46	22		389	259	123	
	9.1 ±1.1	6.4 ± 1.4	3.1 ± 0.6		7.0 ± 0.5	5.0 ±0.4	2. 1± 0.2	
Other race	17	14	11		217	165	66	
	3.4 ±1.2	2.6 ± 0.8	2.0± 0.6		4.0 ± 0.3	3.1 ±0.3	1.3 ± 0.2	
Poverty income ratio				0.08				0.34
<1.000	155	76	56		466	315	144	
	26.4 ±2.3	12.3 ± 1.5	10.1 ± 1.6		10.1 ± 0.6	7.2 ±0.5	3.1 ±0.3	
<1.300	37	34	7		159	106	47	
	8.1 ±1.8	7.0 ± 1.3	1.1 ± 0.4		3.3 ± 0.3	3.0 ±0.3	1.0 ± 0.1	
<1.850	38	31	15		192	136	52	
	7.1 ±1.5	6.0 ± 1.3	3.0 ± 0.8		5.0 ± 0.5	4.0 ±0.3	1.2 ± 0.2	
≥1.850	34	27	10		687	584	225	
	9.3 ±2.0	7.4 ± 1.7	2.2 ± 0.9		28.3 ± 1.1	25.4 ±1.2	8.4 ± 0.6	
SNAP participating^3^				0.86				0.02
Yes	148	89	44		432	244	102	
	38.5 ±2.8	24.1 ± 2.6	12.1 ± 1.9		34.0 ± 1.7	19.2 ±1.3	8.2 ± 1.1	
No	39	28	13		168	134	68	
	12.3 ± 2.2	8.0 ± 1.9	5.0 ± 1.6		17.0 ± 1.4	15.5 ±1.3	6.1 ± 0.9	
NSLP (weekly frequency)^4^				0.58				0.56
0	39	24	14		298	261	100	
	10.6 ±1.9	5.2 ± 1.2	3.0 ± 0.9		12.0 ± 0.9	11.4 ±0.7	4.0 ± 0.5	
1–4	44	29	12		289	206	99	
	8.8 ±1.6	4.6 ± 0.8	2.0 ± 0.5		10.3 ± 0.7	8.0 ±0.6	2.9 ± 0.4	
5	157	100	51		792	591	231	
	32.2 ±2.9	22.4 ± 2.1	11.2 ± 1.5		24.3 ± 1.1	20.1 ±1.2	7.0 ± 0.5	
NSBP (weekly frequency)^5^				0.94				0.38
0	94	60	25		719	554	219	
	22.1 ±2.2	14.0 ± 1.9	6.4 ± 1.6		30.6 ± 1.2	26.3 ±1.2	9.0 ± 0.9	
1–4	47	29	13		203	175	67	
	9.4 ±1.2	6.3 ± 1.2	2. 7± 0.5		7.1 ± 0.6	6.6 ±0.5	2.2 ± 0.4	
5	79	55	32		283	196	100	
	19.0 ±2.6	13.0 ±2.0	7.1 ±1.3		9.0 ±0.5	6.2 ±0.6	3.0 ±0.4	
BMI (kg/m^2^)^6^				0.09				0.002
Underweight	4	3	3		52	49	20	
	1.0 ±0.5	0.3 ± 0.2	0.4 ±0.3		1.4 ±0.2	1.9 ±0.4	1.0 ± 0.2	
Healthy weight	146	94	48		845	711	246	
	7.0 ± 2.3	19.0 ±2.2	9.4 ± 1.5		27.1 ± 0.9	25.2 ±1.1	7.3 ± 0.6	
Overweight	42	37	8		253	185	103	
	9.0 ± 1.4	7.0 ±1.1	1.3 ± 0.4		8.1 ± 0.5	6.0 ±0.4	3.0 ± 0.4	
	72	34	29		354	196	99	
Obesity	14.4 ± 1.7	6.0 ±1.2	5.2 ± 1.0		10.0 ± 0.6	6.0 ±0.5	3.0 ± 0.3	

Abbreviations: NSBP, National School Breakfast Program; NSLP, National School Lunch Program; SE, standard error; SNAP, Supplemental Nutrition Assistance Program.

1 Complex survey design is adjusted according to directions of the Centers for Disease Control and Prevention. Child food security status within the household was used to estimate food security status. Percentages may not add ≤100% due to rounding or missing data for some variables.

2 Rao-Scott F adjusted χ^2^ tests were used to compare characteristics from egg-rich diets by food security status with a 2-sided *P* value of <0.05.

3 Among adolescents 14–17 y old (*n* = 2124 missing).

4 Among adolescents 14–17 y old (*n* = 296 missing).

5 Among adolescents 14–17 y old (*n* = 683).

6 BMI, underweight (less than the 5th percentile), healthy weight (5th percentile to <85th percentile), overweight (85th percentile to <95th percentile), obesity (≥95th percentile).

### Nutrient status and contribution of supplements by food security

No significant differences were observed for the estimated mean usual nutrient intake when stratified by food security status. However, regardless of food security status, >80% of adolescents were at risk of inadequacy for vitamin D and vitamin E. Only 9–14% of adolescents had intakes greater than the AI for choline (Supplemental Table 1).

Despite minimal differences in total usual nutrient intakes by food security status for most nutrients, mean overall TNI scores were statistically significantly lower (TNI score: 65 out of 100) for adolescents with food insecurity when compared with adolescents with food security (TNI score: 70 out of 100; *P* < 0.003). Additionally, TNI component scores for adolescents with food insecurity were significantly lower (*P* < 0.003) compared with adolescents with food security for magnesium (TNI scores: 58 compared with 65 out of 100) and potassium (TNI scores: 72 compared with 78 out of 100) (Supplemental Table 2). No significant differences in mean overall FNI scores were noted; however, similar to the TNI component scores, adolescents with food insecurity scored statistically significantly lower (*P* < 0.003) on the FNI component scores for magnesium (FNI score: 57 compared with 64 out of 100) and potassium (FNI score: 72 compared with 78 out of 100) compared to adolescents with food security (Supplemental Table 2).

### The role of eggs in achieving nutrient adequacy by food security

There were significant differences in the mean usual intake for 7 nutrients (lutein+zeaxanthin, choline, selenium, vitamin D, vitamin B2, DHA, and protein) out of 21 nutrients analyzed when comparing the adolescent groups based on food security status and egg-rich diets (Supplemental Table 3). The food-secure group consuming primarily egg dishes had higher mean usual intakes (*P* < 0.0002) of lutein+zeaxanthin, choline, selenium, vitamin D, vitamin B2, DHA, and protein when compared with other groups that consumed eggs to a lesser extent (e.g., eggs as ingredients in dishes or nonegg consumers) and/or faced food-insecure situations. For example, food-secure adolescents consuming primarily egg dishes had higher mean usual choline intakes (408 mg) when compared with other food-secure adolescents who either consumed eggs as ingredients in dishes (295 mg) or who were nonegg consumers (269 mg), as well as food-insecure adolescents consuming eggs as ingredients in dishes (296 mg) and nonegg consumers (217 mg) (Supplemental Table 3).

Among food-secure and food-insecure adolescents, consuming eggs was related to a lower proportion of the population at risk of inadequate intakes of choline, vitamin A, potassium, folate, calcium, magnesium, vitamin D, iron, vitamin B2, zinc, vitamin E (results should be interpreted cautiously as there are no detectable deficiencies from current dietary intake), vitamin C, and protein. For instance, between 44–70% of adolescents in the different egg-rich diet groups (regardless of food security status) were at risk of inadequate vitamin A intake, with the exception being food-secure adolescents consuming primarily egg dishes (i.e., 39% < EAR). Only 3–23% of adolescents exceeded the AI for choline, yet the estimate was 33% for food-secure adolescents consuming primarily egg dishes (Table 2). Similarly, ∼60–70% of adolescents were at risk of inadequacy for calcium and magnesium, and among the food-insecure group that were nonegg consumers, the risk of inadequacy was >80% for both nutrients. Also, between 10–40% of adolescents did not meet the protein recommendations according to the DGA and between 26–67% according to the TFP.

**TABLE 2:** Estimated prevalence of usual nutrient intakes less than the Estimated Average Requirement, Dietary Guidelines for Americans, and Thrifty Food Plan protein recommendations or above the Adequate Intake among United States adolescents (14–17 y), by household child food security status and egg-rich diets, National Health and Nutrition Examination Survey 2007–2018^1^

Nutrient	Recommendation^2^ (per day)	Food insecure			Food secure		
		Non-egg consumers	Eggs as ingredients in dishes	Primarily egg dishes	Non-egg consumers	Eggs as ingredients in dishes	Primarily egg dishes
		% ± SE (*n* = 264)	% ± SE (*n* = 168)	% ± SE (*n* = 88)	% ± SE (*n* = 1504)	% ± SE (*n* = 1141)	% ± SE (*n* = 468)
Choline, mg^3^	475	2.7 ±2.4	12.1 ±2.0	22.8 ±6.6	7.7 ±1.2	11.2 ±1.3	32.5 ±3.5
Vitamin A, *μ*g	485–630	69.6 ±7.8	49.7 ±4.4	43.5 ±5.1	54.9 ±2.2	52.5 ±3.6	39.2 ±2.6
Potassium, mg^3^	2300–3000	21.5 ±6.5	44.7 ±2.7	39.8 ±4.0	37.3 ±2.4	44.1 ±3.1	49.6 ±3.0
Folate, *μ*g	330	45.3 ±13.5	21.0 ±4.5	20.1 ±5.7	26.2 ±1.6	20.9 ±1.5	22.6 ±2.6
Calcium, mg	1100	83.0 ±8.2	66.8 ±4.3	68.2 ±6.1	70.4 ±1.6	63.2 ±2.2	60.3 ±2.9
Selenium, *μ*g	45	14.8 ±9.4	4.5 ±1.1	2.0 ±1.2	6.1 ± 1.2	3.5 ±0.5	1.5 ±0.3
Magnesium, mg	300–340	87.5 ±8.0	60.9 ±2.9	67.2 ±4.4	71.4 ±1.8	62.4 ±2.7	59.5 ±2.7
Vitamin D, *μ*g	10	95.2 ±1.4	90.5 ±1.5	88.8 ±4.1	91.2 ±1.0	89.8 ±1.0	84.1 ±1.4
Iron, mg	7.7–7.9	25.1 ±7.9	9.4 ±2.3	12.5 ±2.4	15.3 ±1.8	8.4 ±0.7	8.8 ±1.3
Zinc, mg	7.3–8.5	44.9 ±12.0	28.6 ± 3.5	24.8 ±5.4	32.2 ±3.2	23.8 ±2.3	23.2 ±2.0
Vitamin E, mg	12	95.8 ±2.5	82.4 ±2.8	78.5 ±6.0	88.6 ±1.4	80.1 ±1.9	81.3 ±2.7
Vitamin B12, *μ*g	2	15.0 ±2.6	13.2 ±1.9	11.7 ±3.5	15.8 ±1.7	11.9 ±1.2	10.5 ±1.2
Vitamin B2, mg	0.9–1.1	23.6 ±11.2	8.3 ±1.6	7.2 ±2.2	12.4 ±1.3	8.0 ±0.9	4.9 ±0.7
Vitamin B1, mg	0.9–1	30.5 ±9.4	14.3 ±2.4	14.1 ±4.6	18.0 ±1.7	11.3 ±1.0	11.3 ±1.9
Vitamin B6, mg	1–1.1	18.5 ±2.8	13.7 ±2.1	15.4 ±3.0	17.3 ±1.6	12.0 ±1.7	10.3 ±1.5
Vitamin C, mg	56–63	61.3 ±6.3	40.7 ± 2.6	47.6 ±4.4	46.3 ±5.2	47.9 ±2.5	39.1 ±3.5
Vitamin B3, mg	11–12	11.9 ±2.8	6.7 ±1.2	7.5 ±3.0	9.1 ±1.0	5.8 ±1.0	6.4 ±0.8
Protein DGA, g	46–52	40.0 ±16.6	15.8 ±2.8	14.4 ±4.1	22.0 ±2.6	14.4 ±2.1	9.8 ±1.2
Protein TFP, g	55–75	66.7 ±14.9	34.9 ±4.1	34.2 ±6.2	44.7 ±3.3	34.0 ±3.1	26.3 ±2.1

Abbreviations: AI, Adequate Intake, DFE, dietary folate equivalents; DGA, Dietary Guidelines for Americans; EAR, Estimated Average Requirement; RAE, retinol activity equivalents; SE, standard error; TFP, Thrifty Food Plan.

1 Child food security status within the household was used to estimate food security status; Estimations of vitamin A as RAE, Folate as DFE, and vitamin E as alpha-tocopherol equivalents.

2 Recommendation column shows the Dietary Reference Intakes: EAR or AI, and the protein recommendation according to the DGA or TFP. EAR, AI, DGA, and TFP ranges for adolescents 14–18 y are dependent on sex.

3 Estimated using the AI as an EAR is not established.

### The role of eggs and supplements in achieving nutrient adequacy by food security

TNI and FNI component scores for magnesium, potassium, choline, and vitamin D showed greater gaps between adolescents with food insecurity who were nonegg consumers and adolescents with food security who consumed primarily egg dishes (*P* < 0.0002). One example of this pattern is that adolescents experiencing food insecurity who did not consume eggs scored lower on the TNI (54.6 out of 100) and FNI (54 out of 100) for magnesium (*P* < 0.0002) compared to their food-secure counterparts who consumed primarily egg dishes (TNI score: 72 out of 100; FNI score: 70 out 100) (Supplemental Table 4).

### Nutrient status with the addition of 1 egg by food security

Among adolescents with food security who consumed primarily egg dishes, there was an increase of 128 mg (*P* < 0.0004) in mean usual choline intake (537 mg) with the addition of an egg, increasing the estimate above the AI (475 mg) for choline. Similar increases for mean choline were observed with the addition of an egg among other egg-rich diet groups; however, those increases were not enough to increase the mean above the recommended AI. In addition to choline, significant increases in mean usual vitamin D intake were also observed among food-secure adolescents, with the addition of an egg among nonegg consumers (an increase of 1.3 *μ*g) and consumers of eggs as ingredients in dishes (an increase of 1.1 *μ*g) (*P* < 0.0004) (Supplemental Table 5).

The addition of an egg showed an improvement in the proportion of adolescents meeting the recommendations for choline, vitamin A, folate, iron, and vitamin B2. For instance, the proportion of adolescents with AI for choline increased from ∼3–33% to 22–63%. Similarly, for vitamin A, the addition of an egg decreased the inadequate intake estimate from 39% to 29% for the food-secure group consuming primarily egg dishes and from 70% to 52% for the food-insecure group that were nonegg consumers (Table 3). Finally, for folate (from 45% to 35%), iron (from 25% to 18%), and vitamin B2 (from 24% to 10%), the addition of 1 egg reduced the inadequate intake estimate among food-insecure adolescents who were nonegg consumers.

**TABLE 3:** Estimated prevalence of usual nutrient intakes, with the addition of 1 egg^1^, less than the Estimated Average Requirement, Dietary Guidelines for Americans and Thrifty Food Plan protein recommendations or above the Adequate Intake among United States adolescents (14–17 y), by household child food security status and egg-rich diets, National Health and Nutrition Examination Survey 2007–2018^2^

Nutrient	Recommendation^3^ (per day)	Food insecure			Food secure		
		Non-egg consumers	Eggs as ingredients in dishes	Primarily egg dishes	Non-egg consumers	Eggs as ingredients in dishes	Primarily egg dishes
		% ± SE (*n* = 264)	% ± SE (*n* = 168)	% ± SE (*n* = 88)	% ± SE (*n* = 1504)	% ± SE (*n* = 1141)	% ± SE (*n* = 468)
Choline, mg^4^	475	21.7 ±5.4	36.2 ±4.3	48.5 ±8.5	29.7 ±2.6	35.5 ±2.2	63.4 ± 3.7
Vitamin A, *μ*g	485–630	52.2 ±5.2	38.8 ±4.4	34.5 ± 4.9	41.9 ± 2.1	39.6 ± 3.3	28.6 ± 2.5
Potassium, mg^4^	2300–3000	24.6 ±6.5	46.2 ±2.9	39.9 ±4.0	40.0 ±2.5	47.2 ± 3.2	52.3 ± 3.1
Folate, *μ*g	330	34.9 ±9.4	16.8 ±4.1	15.9 ±5.2	21.2 ± 1.5	16.8 ± 1.4	18.1 ± 2.3
Calcium, mg	1100	80.4 ±8.0	65.0 ±4.4	66.4 ± 6.2	68.5 ± 1.6	61.4 ± 2.2	58.3 ± 2.9
Selenium, *μ*g	45	2.8 ±1.6	0.9 ±0.2	0.3 ±0.2	1.3 ± 0.3	0.6 ± 0.1	0.2 ± 0.0
Magnesium, mg	300–340	84.0 ±7.3	59.3 ± 2.9	66.5 ±4.5	69.6 ±1.9	60.4 ± 2.7	57.5 ± 2.7
Vitamin D, *μ*g	10	92.5 ±1.6	88.5 ±1.7	84.9 ±4.5	88.7 ± 1.1	87.7 ± 1.1	82.5 ± 1.4
Iron, mg	7.7–7.9	17.9 ±6.0	6.1 ±1.7	8.4 ±1.8	10.5 ± 1.4	5.6 ± 0.5	5.7 ± 1.0
Zinc, mg	7.3–8.5	34.7 ±8.9	23.4 ± 3.3	20.4 ±4.9	25.9 ± 2.9	18.8 ± 2.0	18.3 ± 1.7
Vitamin E, mg	12	93.6 ±2.3	80.2 ±3.1	75.9 ±6.5	86.8 ± 1.5	78.0 ± 1.9	79.1 ± 2.9
Vitamin B12, *μ*g	2	8.4 ±1.7	7.8 ±1.2	6.7 ±2.2	9.4 ± 1.1	7.1 ± 0.8	6.2 ± 0.8
Vitamin B2, mg	0.9–1.1	9.9 ±3.9	4.1 ±0.9	3.7 ± 1.2	6.3 ± 0.8	3.9 ± 0.5	2.2 ± 0.3
Vitamin B1, mg	0.9–1	27.3 ±8.7	12.9 ±2.2	12.9 ±4.4	16.0 ±1.6	9.9 ± 0.9	9.9 ± 1.7
Vitamin B6, mg	1–1.1	16.9 ±2.7	12.7 ±2.0	14.6 ±2.9	15.8 ±1.5	10.9 ± 1.6	9.4 ± 1.4
Vitamin C, mg	56–63	61.1 ±6.3	40.9 ±2.6	48.1 ±4.3	46.3 ± 5.2	47.8 ± 2.5	39.1 ± 3.5
Vitamin B3, mg	11–12	11.7 ±2.8	6.7 ±1.2	7.7 ± 3.1	9.1 ± 1.0	5.8 ± 1.0	6.3 ± 0.9
Protein DGA, gm	46–52	23.4 ±8.3	9.5 ±2.1	8.5 ±2.9	13.9 ± 2.0	8.6 ± 1.4	5.5 ± 0.8
Protein TFP, gm	55–75	48.9 ±9.9	27.5 ±4.0	28.2 ±6.0	35.3 ± 3.2	25.8 ± 2.8	19.2 ± 1.9

Abbreviations: AI, Adequate Intake; DFE, dietary folate equivalents; DGA, Dietary Guidelines for Americans; EAR, Estimated Average Requirement; RAE, retinol activity equivalents; SE, standard error; TFP, Thrifty Food Plan.

1 One serving size of 50 g of an “Egg, whole, boiled or poached” from the most current version of the Food and Nutrient Database for Dietary Studies 2019–2020 [28].

2 Household with children was used to estimate food security status. Child food security status within the household was used to estimate food security status; Estimations of vitamin A as RAE. The percentage ± SE are prevalence of adolescents not meeting the recommended nutrient intake, which means a percentage less than the EAR, DGA, and TFP protein recommendations or a percentage above the AI. Estimations of vitamin A as RAE, Folate as DFE, and vitamin E as alpha-tocopherol equivalents.

3 Recommendation column shows the Dietary Reference Intakes: EAR, or AI, and the protein recommendation according to the DGA or TFP. EAR, AI, DGA, and TFP ranges for adolescents 14–18 y are dependent on sex.

4 Estimated using the AI as an EAR is not established.

### Nutrient status with the addition of both supplements and 1 egg by food security

The addition of an egg also showed increases in TNI and FNI component scores for choline from 46–78 to 74–93 out of 100 for the TNI and from 46–78 to 73–93 out of 100 for the FNI (*P* < 0.0005) across all food-secure/egg-rich diet groups. The addition of 1 egg also demonstrated an increase in the TNI (7-point increase) and FNI (8-point increase) component scores for vitamin D among adolescents experiencing food insecurity who consumed primarily egg dishes and an increase in the FNI score (8-point increase) among those living in food-secure situations which included eggs as ingredients in dishes (Supplemental Table 6).

## Discussion

United States adolescents were at high risk of inadequate intake of several nutrients, including choline, potassium, calcium, magnesium, vitamin D, vitamin E, and vitamin C, regardless of population subgroup. It is important to indicate that there have been suggestions to reassess the EAR for vitamin E because, despite the widespread occurrence of insufficient vitamin E intake, visible clinical symptoms of deficiency are rare among United States populations [48,49]. Significant differences were not observed when comparing usual nutrient intake among adolescents by food security status alone, indicating pervasive nutrient inadequacy across all food security groups, similar to previous reports [7,8,50].

However, when examining usual nutrient intake across different groups based on food security status and egg-rich diets, results show, in general, higher usual nutrient intake for adolescents experiencing food security who include primarily egg dishes, a potential reason for why this group had higher usual nutrient intakes for lutein + zeaxanthin, choline, selenium, vitamin D, vitamin B2, DHA, and protein when compared with the other food security/egg-rich diet subgroups. For both food security groups, those consuming primary egg dishes had a higher proportion of nutrient adequacy compared with those not consuming eggs and similarly those consuming eggs as ingredients had higher nutrient adequacy compared with those not consuming eggs (except for vitamin C among food secure adolescents). The proportion with inadequacy was lower in both food secure and insecure groups for several nutrients among the adolescents who consumed primarily egg dishes, followed by those who consumed eggs as ingredients in dishes, showing that even consuming eggs as ingredients in dishes may offer a promising opportunity to increase nutrient intake. Adding 1 egg/day reduced the proportion of the group with nutrient inadequacy for several nutrients, varying by food security status and egg-consuming group status, regardless of which metric was used. These findings suggest that during adolescence, where there is a compromised intake of some nutrients that can be exacerbated by food-insecure situations, egg consumption is associated with less risk of nutrient inadequacy.

Overall, usual nutrient intake and adherence to nutrient recommendations were poor among United States adolescents. Prior investigations show that adolescents have different times of eating occasions (late night) and have different snack patterns that contribute to saturated fat, added sugars, and sodium [51,52]. This difficulty in achieving a balanced and nutrient-rich diet poses implications for the health and well-being of this demographic group, potentially leading to increased risk for obesity, chronic diseases, and compromised cognitive development. Addressing and improving the dietary patterns of United States adolescents is important for health promotion in both short and long-term lifestyle behaviors (e.g., physical activity, frequency of meals, meals during television viewing) and quality of life [53]. The results of this study support prior work in this life stage and further suggest that nutrient gaps can be ameliorated using nutrient-rich foods; while eggs were used here, other foods could be evaluated in future research. Understanding food and nutrition security among this high-risk population is apposite to improving public health.

Eggs as ingredients in dishes represent a unique opportunity to increase nutrient intakes shown in these results. Food-secure adolescents who included eggs in dishes had a lower risk of vitamin A inadequacy and higher TNI (magnesium, potassium, and folate) and FNI (magnesium) mean component scores compared to food-insecure adolescents who were nonegg consumers. Also, in the results shown here, the addition of an egg demonstrated a higher prevalence of adolescents with intakes above the AI for choline compared with a previously published study [54]. A prior NHANES study [54] modeled an additional 7 eggs/wk (or 1 egg/day) to the current dietary pattern, resulting in 23% of children aged 9–18 y being above the AI for choline [54]. In this study with the addition of 1 egg, intakes above the AI for choline were observed for 63% of adolescents aged 14–17 y who were food secure, consuming primarily egg dishes, 49% of adolescents with food insecurity consuming primarily egg dishes, and 36% of adolescents with food insecurity consuming eggs as ingredients in dishes. The differences here, compared with previous literature, highlight the importance of considering age, food security status, and egg-rich diets to mean usual intakes when modeling the addition of an egg, specifically when evaluating mean choline intake.

As with all cross-sectional analyses, this study has several strengths and limitations. Strengths of this study include the examination of data derived from a nationally representative sample of United States adolescents. The combination of 6 y of NHANES allowed a large sample size to generate reliable estimates. However, the sample size among food-insecure adolescents was still relatively small and might have led to larger SEs and a reduced capacity to identify statistical significance. The random measurement errors inherent to 24-h recalls were addressed by estimating total usual intakes through the use of the NCI method. Nonetheless, potential systematic measurement errors linked to food security status remain a plausible source of bias when comparing subgroups affected by this situation. Additionally, using a single 24-h recall to estimate egg-rich diet levels may entail an error in representing the daily egg intake; hence, future studies might use other methods to estimate egg consumption behavior. Also, to preserve the stability of the estimates, the usual intake and nutrient exposure scores were not stratified by sex. As a result, the mean usual intake estimates for protein may potentially reflect the influence of lower protein intakes in girls than boys. Furthermore, the proportion of adolescents not meeting the minimum protein recommendation varies between the DGA and TFP. The TFP’s higher calorie requirement raises the protein threshold, which likely explains why a greater percentage of adolescents fail to meet the protein recommendation compared to the DGA. This highlights how calorie intake assumptions can affect macronutrient adequacy evaluations.

In conclusion, most United States adolescents as a group failed to meet many micronutrient and protein recommendations and presented a high risk of inadequacy for several nutrients, including choline, potassium, calcium, magnesium, vitamin C, vitamin D, and vitamin E, and protein (for the TFP). Food-secure adolescents that consumed primarily egg dishes had higher nutrient intakes for lutein + zeaxanthin, choline, selenium, vitamin D, vitamin B2, and DHA and compliance with protein recommendations when compared with other food security and egg-rich diet subgroups. Food-secure and -insecure adolescents who consumed eggs as ingredients in dishes showed higher nutrient adequacy than nonegg consumers. Including a daily egg in the diet enhanced choline and vitamin D intake, as well as the overall micronutrient intake patterns among the food security and egg-rich diet subgroups, as indicated by the TNI and FNI scores. Efforts aimed at enhancing the accessibility and availability of nutrient-rich foods are crucial in lessening the nutrition risk for all adolescents and considering the prevalence of food and nutrition insecurity, more tailored strategies may be needed.

## Author contributions

The authors’ responsibilities were as follows – AM-J, AEC-P, RLB, HAE-M: designed research; AM-J: analyzed data and wrote the article; AEC-P, RLB, HAE-M: critically reviewed and edited the manuscript; HAE-M: had primary responsibility for final content; and all authors: read and approved the final manuscript.

## Funding

This research was supported by the American Egg Board’s Egg Nutrition Center, Contract number 22057732. HAE-M received support from the Danone International Prize for Alimentation from the Danone Institute International Association and the French Foundation for Medical Research.

## Data availability

Data described in the article and codebook are publicly and freely available without restriction at https://www.cdc.gov/nchs/nhanes/index.htm. Analytic code will be made available upon request pending application and approval.

## Conflict of interest

Unrelated to this work, AEC-P currently serves on the board of editors for the Journal of the Academy of Nutrition and Dietetics. RLB has served as a consultant in the past to Nestlé and the Think Healthy Group and is a trustee of the International Food Information Council. RLB has received travel support to present her research on dietary supplements and is an editor for the Journal of Nutrition and played no role in the journal’s evaluation of the manuscript. HAE-M currently serves on the board of editors for the Journal of the Academy of Nutrition and Dietetics and Advances in Nutrition. AM-J has no conflicts of interest to disclose.

## References

[1] Economic Research Service (2023). U.S. Department of Agriculture: Definitions of food security. https://www.ers.usda.gov/topics/food-nutrition-assistance/food-security-in-the-u-s/definitions-of-food-security/.

[2] US Department of Agriculture (2020). US Department of Health and Human Services: Dietary guidelines for Americans-2020-2025. https://www.dietaryguidelines.gov/sites/default/files/2021-03/Dietary_Guidelines_for_Americans-2020-2025.pdf.

[3] US Department of Agriculture (2020). https://www.dietaryguidelines.gov/sites/default/files/2020-07/ScientificReport_of_the_2020DietaryGuidelinesAdvisoryCommittee_first-print.pdf.

[4] Eicher-Miller H., Park C., Bailey R. (2015). Dietary supplements in health promotion.

[5] Cowan A.E., Tooze J.A., Gahche J.J., Eicher-Miller H.A., Guenther P.M., Dwyer J.T. (2023). Trends in overall and micronutrient-containing dietary supplement use in US adults and children, NHANES 2007-2018. J Nutr.

[6] Jun S., Cowan A.E., Tooze J.A., Gahche J.J., Dwyer J.T., Eicher-Miller H.A. (2018). Dietary supplement use among U.S. Children by family income, food security level, and nutrition assistance program participation status in 2011⁻2014. Nutrients.

[7] Eicher-Miller H.A., Zhao Y. (2018). Evidence for the age-specific relationship of food insecurity and key dietary outcomes among US children and adolescents. Nutr. Res. Rev..

[8] Jun S., Cowan A.E., Dodd K.W., Tooze J.A., Gahche J.J., Eicher-Miller H.A. (2021). Association of food insecurity with dietary intakes and nutritional biomarkers among US children, National Health and Nutrition Examination Survey (NHANES) 2011-2016. Am. J Clin. Nutr..

[9] Eicher-Miller H.A., Boushey C.J., Bailey R.L., Yang Y.J. (2020). Frequently consumed foods and energy contributions among food secure and insecure U.S. children and adolescents. Nutrients.

[10] Egg Nutrition Center, Full Nutrition Facts for 1 large egg (50g) (2019). [cited March 15, 2024]. Available from: https://www.incredibleegg.org/wp-content/uploads/2023/07/large-vertical.pdf

[11] US Department of Agriculture (2021). Thrifty food plan, 2021. Food and Nutrition Service.

[12] Agricultural Research Service. What we eat in America Food Categories; 2017-March 2020. Prepandemic [Internet]; [cited June 20, 2023]. Available from: https://www.ars.usda.gov/ARSUserFiles/80400530/pdf/1720/Food_Categories_2017-March%202020%20Prepandemic.pdf.

[13] Papanikolaou Y., Fulgoni V.L. (2021). Modeling the removal and addition of eggs in the current US diet is linked to choline and lutein + zeaxanthin usual intakes in childhood. Curr. Dev. Nutr..

[14] Papanikolaou Y., Fulgoni V.L. (2021). Increasing egg consumption at breakfast is associated with increased usual nutrient intakes: A modeling analysis using NHANES and the USDA child and adult care food program school breakfast guidelines. Nutrients.

[15] Zipf G., Chiappa M., Porter K.S., Ostchega Y., Lewis B.G., Dostal J. (2013). National Health and Nutrition Examination Survey: plan and operations, 1999–2010. Vital Health Stat.

[16] National Center for Health Statistics (2022). NCHS ethics review board (ERB) approval∗. https://www.cdc.gov/nchs/nhanes/irba98.htm.

[17] Johnson C.L., Dohrmann S.M., Burt V.L., Mohadjer L.K. (2014). National health and nutrition examination survey: sample design, 2011-2014. Vital Health Stat.

[18] US Department of Health and Human Services, Poverty guidelines [Internet]; [cited October 20, 2023]. Available from: https://aspe.hhs.gov/topics/poverty-economic-mobility/poverty-guidelines.

[19] Toossi S., Jones J.W. (2023). https://www.ers.usda.gov/webdocs/publications/106763/eib-255.pdf?v%3d8994.7.

[20] Ogden C.L., Flegal K.M. (2010). Changes in terminology for childhood overweight and obesity. Natl. Health Stat. Rep..

[21] Bickel G., Nord M., Price C., Hamilton W., Cook J. (2000). https://fnsprod.azureedge.us/sites/default/files/FSGuide.pdf.

[22] National Center for Health Statistics (2021). National Health and Nutrition Examination Survey 2017-2018 data documentation, codebook, and frequencies. Food security.

[23] Jun S., Zeh M.J., Eicher-Miller H.A., Bailey R.L. (2019). Children’s dietary quality and micronutrient adequacy by food security in the household and among household children. Nutrients.

[24] Nord M., Hopwood H. (2007). Recent advances provide improved tools for measuring children’s food security. J Nutr.

[25] Nord M., Bickel G. (2002). https://www.ers.usda.gov/webdocs/publications/46613/31444_fanrr25_002.pdf?v%3d6958.4.

[26] Blanton C.A., Moshfegh A.J., Baer D.J., Kretsch M.J. (2006). The USDA Automated Multiple-Pass Method accurately estimates group total energy and nutrient intake. J Nutr.

[27] Agricultural Research Service. AMPM - USDA Automated Multiple-Pass Method [Internet]. [updated Jun 1, 2021; cited March 31, 2024]. Available from: https://www.ars.usda.gov/northeast-area/beltsville-md-bhnrc/beltsville-human-nutritionresearch-center/food-surveys-research-group/docs/ampm-usda-automated-multiple-passmethod/#what.

[28] Cowan A.E., Bailey R.L., Jun S., Dodd K.W., Gahche J.J., Eicher-Miller H.A. (2022). The total nutrient index is a useful measure for assessing total micronutrient exposures among US adults. J Nutr.

[29] Food and Nutrient database for dietary studies [Internet], Agricultural Research Service (2007–2018) [cited October 20, 2023]. Available from: https://www.ars.usda.gov/northeast-area/beltsville-md-bhnrc/beltsville-human-nutrition-research-center/food-surveys-research-group/docs/fndds-download-databases/.

[30] Gahche J.J., Bailey R.L., Potischman N., Ershow A.G., Herrick K.A., Ahluwalia N. (2018). Federal monitoring of dietary supplement use in the resident, civilian, noninstitutionalized US population, national health and nutrition examination survey. J Nutr.

[31] Dietary supplement database: Product information (DSPI) [Internet] (2014). https://wwwn.cdc.gov/Nchs/Nnyfs/DSPI.htm.

[32] Agricultural Research Service (2020). Food data central search results. Egg, whole, boiled or poached [Internet]. FNDDS 2019-2020. https://fdc.nal.usda.gov/fdc-app.html#/food-details/1100185/nutrients.

[33] Tooze J.A., Midthune D., Dodd K.W., Freedman L.S., Krebs-Smith S.M., Subar A.F. (2006). A new statistical method for estimating the usual intake of episodically consumed foods with application to their distribution. J Am. Diet Assoc..

[34] National Academies of Sciences (2019).

[35] Institute of Medicine (US) (2011).

[36] Institute of Medicine (US) (2000).

[37] Institute of Medicine (US) (2001).

[38] Institute of Medicine (US) Standing Committee on the Scientific Evaluation of Dietary Reference Intakes (1997).

[39] Institute of Medicine (US) (2005).

[40] Institute of Medicine (US) (1998).

[41] Institute of Medicine (US) (2000).

[42] Rao J.N., Shao J. (1999). Modified balanced repeated replication for complex survey data. Biometrika.

[43] Burt V.L., Cohen S.B. (1984). A comparison of methods to approximate standard errors for complex survey data. Rev. Public Data Use..

[44] National Academy of Sciences (2023).

[45] Yang L., Cao C., Kantor E.D., Nguyen L.H., Zheng X., Park Y. (2019). Trends in sedentary behavior among the US population, 2001-2016. JAMA.

[46] Cowan A.E., Jun S., Tooze J.A., Dodd K.W., Gahche J.J., Eicher-Miller H.A. (2023). A narrative review of nutrient based indexes to assess diet quality and the proposed total nutrient index that reflects total dietary exposures. Crit. Rev. Food Sci. Nutr..

[47] Centers for Disease Control and Prevention, National Center for Health Statistics, Dietary interview–total nutrient intakes, first day 2017–2018 (DR1TOT_J) [cited June 21, 2023]. Available from: https://wwwn.cdc.gov/nchs/nhanes/search/datapage.aspx?Component=Dietary&CycleBeg.

[48] Traber M.G. (2014). Vitamin E inadequacy in humans: causes and consequences. Adv. Nutr..

[49] MacFarlane A.J., Cogswell M.E., de Jesus J.M., Greene-Finestone L.S., Klurfeld D.M., Lynch C.J. (2019). A report of activities related to the Dietary Reference Intakes from the Joint Canada-US Dietary Reference Intakes Working Group. Am. J Clin. Nutr..

[50] Kirkpatrick S.I., Dodd K.W., Parsons R., Ng C., Garriguet D., Tarasuk V. (2015). Household food insecurity is a stronger marker of adequacy of nutrient intakes among Canadian compared to American youth and adults. J Nutr.

[51] Tripicchio G.L., Bailey R.L., Davey A., Croce C.M., Fisher J.O. (2023). Snack frequency, size, and energy density are associated with diet quality among US adolescents. Public Health Nutr.

[52] Bailey R.L., Leidy H.J., Mattes R.D., Heymsfield S.B., Boushey C.J., Ahluwalia N. (2022). Frequency of eating in the US population: A narrative review of the 2020 dietary guidelines advisory committee report. Curr. Dev. Nutr..

[53] Lipsky L.M., Nansel T.R., Haynie D.L., Liu D., Li K., Pratt C.A. (2017). Diet quality of US adolescents during the transition to adulthood: changes and predictors. Am. J Clin. Nutr..

[54] Papanikolaou Y., Fulgoni V.L. (2019). Egg consumption in U.S. Children is Associated with Greater Daily Nutrient Intakes, including Protein, lutein + zeaxanthin, choline, α-linolenic acid, and docosahexanoic acid. Nutrients.

