# Supplementary material for: Eating Egg-Rich Diets and Modeling the Addition of One Daily Egg Reduced Risk of Nutrient Inadequacy among United States Adolescents with and without Food Insecurity
Source: J Nutr. 2024 Sep 21;154(11):3475–84. doi: 10.1016/j.tjnut.2024.09.019 (PMC11827025; doi:10.1016/j.tjnut.2024.09.019)
Supplement: Multimedia component 1 [file mmc1.docx]

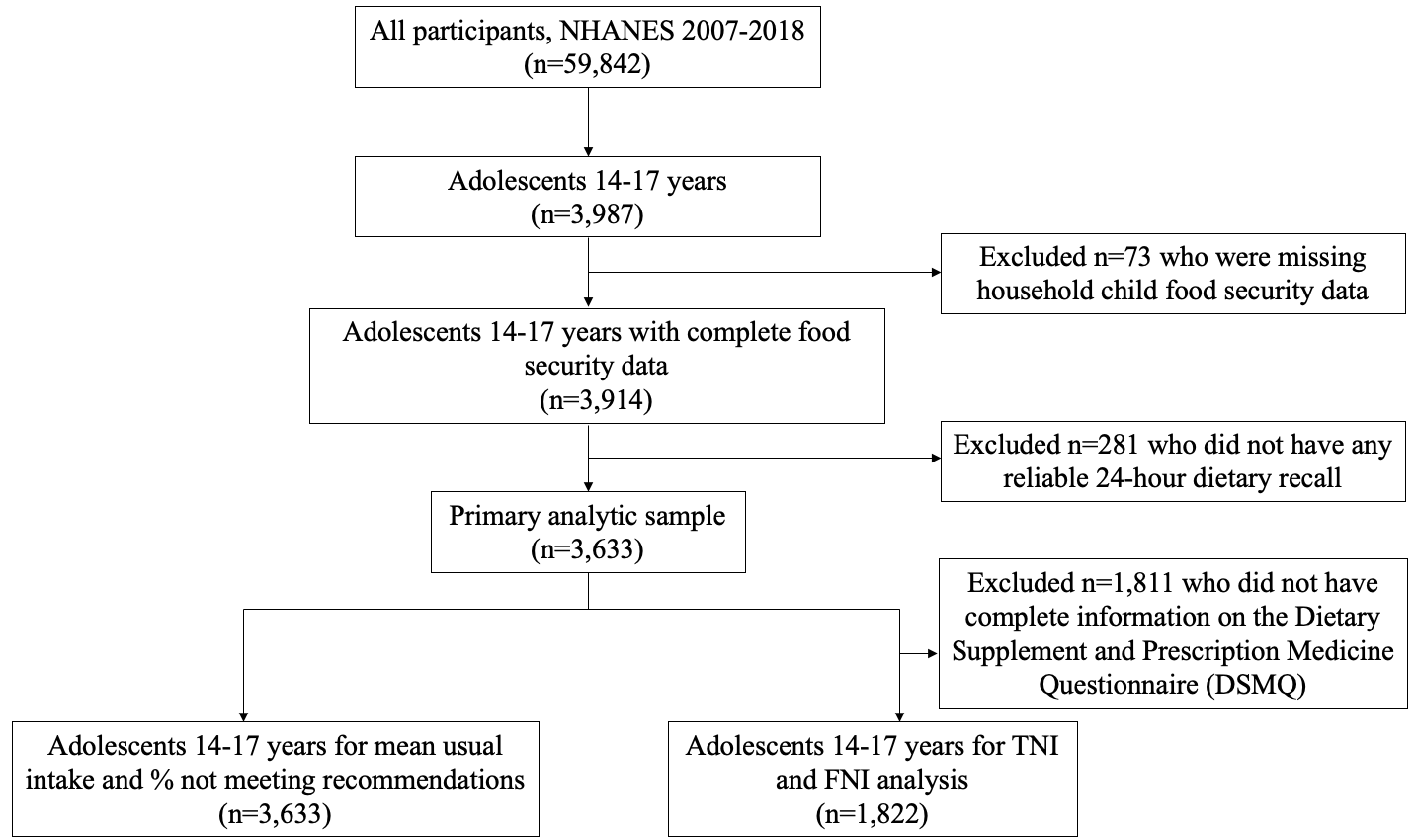


Supplementary Figure 1. Flow chart showing sample identification in the current investigation using data from the National Health and Nutrition Examination Survey 2007-2018

Supplementary Table 1. Comparison of mean and prevalence of the usual nutrient intakes below the Estimated Average Requirement or above the Adequate Intake among U.S. adolescents (14-17 y), by household child food security status, National Health and Nutrition Examination Survey 2007-2018^1*^

|  |  | Food Insecure (n=520) | |  | Food Secure (n=3113) | |
| --- | --- | --- | --- | --- | --- | --- |
| Nutrient | Recommendation^2^  (per day) | Mean $\pm$ SE | % $\pm$ SE |  | Mean $\pm$ SE | % $\pm$ SE |
| Lutein + Zeaxanthin, mcg | — | 1044.5 $\pm$ 94.5 | — |  | 1191.1 $\pm$46.7 | — |
| Choline, mg^3^ | 400-550 | 270.0 $\pm$ 24.9 | 9.2 $\pm$3.1 |  | 305.2 $\pm$6.9 | 13.8 $\pm$1.1 |
| Vitamin A, mcg | 485-630 | 567.0 $\pm$ 42.9 | 57.8 $\pm$4.6 |  | 629.3 $\pm$16.2 | 51.1 $\pm$1.7 |
| Potassium, mg^3^ | 2,300-3,000 | 2309.8 $\pm$113.4 | 32.5 $\pm$4.1 |  | 2564.0 $\pm$43.2 | 42.0 $\pm$1.6 |
| Folate, mcg | 330 | 468.5 $\pm$ 41.5 | 31.7 $\pm$6.3 |  | 525.0 $\pm$ 9.2 | 23.8 $\pm$1.2 |
| Calcium, mg | 1100 | 869.3 $\pm$ 71.4 | 75.4 $\pm$5.6 |  | 984.9 $\pm$15.0 | 66.1 $\pm$1.2 |
| Selenium, mcg | 45 | 96.4 $\pm$ 8.9 | 7.9 $\pm$ 3.0 |  | 109.5 $\pm$2.4 | 4.3 $\pm$0.5 |
| Magnesium, mg | 300-340 | 252.1 $\pm$ 20.6 | 76.6 $\pm$5.9 |  | 286.7 $\pm$ 3.8 | 66.1 $\pm$1.2 |
| Vitamin D, mcg | 10 | 4.3 $\pm$ 0.2 | 92.6 $\pm$1.2 |  | 5.0 $\pm$0.1 | 89.3 $\pm$0.8 |
| Iron, mg | 7.7-7.9 | 13.4 $\pm$0.7 | 16.8 $\pm$3.4 |  | 14.8 $\pm$ 0.2 | 11.6 $\pm$0.8 |
| Zinc, mg | 7.3-8.5 | 10.2 $\pm$0.7 | 35.0 $\pm$5.7 |  | 11.2 $\pm$ 0.2 | 27.5 $\pm$1.6 |
| Vitamin E, mg | 12 | 6.9 $\pm$ 0.5 | 89.9 $\pm$2.7 |  | 8.0 $\pm$ 0.1 | 84.3 $\pm$1.0 |
| Vitamin B12, mcg | 2 | 5.0 $\pm$ 0.2 | 13.8 $\pm$ 1.7 |  | 5.1 $\pm$ 0.1 | 13.4 $\pm$0.9 |
| Vitamin B2, mg | 0.9-1.1 | 1.8 $\pm$ 0.1 | 14.1 $\pm$4.1 |  | 2.0 $\pm$ 0.0 | 9.4 $\pm$0.7 |
| Vitamin B1, mg | 0.9-1 | 1.4 $\pm$ 0.0 | 21.1 $\pm$4.0 |  | 1.6 $\pm$ 0.0 | 14.4 $\pm$0.9 |
| Vitamin B6, mg | 1-1.1 | 1.9 $\pm$ 0.0 | 16.3 $\pm$1.5 |  | 2.0 $\pm$ 0.0 | 14.0 $\pm$1.1 |
| DHA, mg | — | 40.0 $\pm$0.0 | — |  | 40.0 $\pm$0.0 | — |
| Vitamin C, mg | 56-63 | 72.9 $\pm$ 5.2 | 52.1 $\pm$3.6 |  | 82.6 $\pm$ 4.1 | 45.5 $\pm$2.8 |
| Vitamin B3, mg | 11-12 | 24.0 $\pm$ 0.9 | 9.0 $\pm$1.4 |  | 25.2 $\pm$ 0.5 | 7.4 $\pm$0.7 |
| Protein DGA, g | 46-52 | 69.2 $\pm$ 6.7 | 26.3 $\pm$7.5 |  | 78.8 $\pm$ 1.7 | 16.9 $\pm$1.4 |
| Protein TFP, g | 55-75 | 69.2 $\pm$ 6.7 | 49.3 $\pm$8.6 |  | 78.8 $\pm$ 1.7 | 37.5 $\pm$2.0 |

^1^Child food security status within the household was used to estimate food security status. DHA, Docosahexaenoic acid; DGA, Dietary Guidelines for Americans; TFP, Thrifty Food Plan; — no official Dietary Reference Intakes (DRI) (data not shown). Estimations of Vitamin A as RAE, Retinol Activity Equivalents; Folate, as DFE, Dietary Folate Equivalents; and Vitamin E, as alpha tocopherol equivalents

^2^Recommendation column shows the DRI: Estimated Average Requirement (EAR) or Adequate Intake (AI), and the protein recommendation according to the DGA or TFP. EAR, AI, DGA and TFP ranges for adolescents 14-18 years are dependent on sex.

^3^Estimated using the AI as an EAR is not established.

*No significant differences between the food insecure and the food secure groups based on t-test, p< 0.002 to adjust for multiple comparisons using Bonferroni method. Two groups were analyzed, resulting in 1 possible pairwise comparison, calculated as 2!/(2-2)!2. With 21 nutrient markers, this led to a total of 21 (1 by 21) comparisons that were utilized in the Bonferroni adjustment of 0.05/21 for the *P* value=0.002

Supplementary Table 2. Comparison of means of the Food Nutrient Index (FNI) and Total Nutrient Index (TNI) and component scores among U.S. adolescents (14-17 y), by household child food security status, National Health and Nutrition Examination Survey 2007-2018^1*^

| TNI Components | Food Insecure | Food Secure |
| --- | --- | --- |
|  | Mean $\pm$SE  (n=515) | Mean $\pm$SE  (n=1307) |
| Calcium | 66.4 $\pm$1.7 | 71.9 $\pm$1.2 |
| Magnesium | 58.0 $\pm$1.3^a^ | 65.1 $\pm$1.1^a^ |
| Potassium | 72.2 $\pm$1.4^a^ | 78.1 $\pm$1.2^a^ |
| Zinc | 83.0 $\pm$1.2 | 85.6 $\pm$ 0.9 |
| Choline | 53.7 $\pm$1.4 | 56.0 $\pm$ 1.1 |
| Folate, DFE^2^ | 84.9 $\pm$1.6 | 90.0 $\pm$ 0.8 |
| Vitamin C | 67.0 $\pm$2.4 | 72.6 $\pm$ 1.5 |
| Vitamin D | 35.7 $\pm$1.7 | 42.5 $\pm$ 1.5 |
| Total TNI Score | 65.1 $\pm$1.3^a^ | 70.2 $\pm$0.9^a^ |
| FNI Components | Food Insecure | Food Secure |
|  | Mean $\pm$SE  (n=515) | Mean $\pm$ SE  (n=1307) |
| Calcium | 65.0 $\pm$1.7 | 70.8 $\pm$1.2 |
| Magnesium | 57.2 $\pm$1.3^a^ | 64.0 $\pm$ 1.1^a^ |
| Potassium | 72.2 $\pm$1.4^a^ | 78.0 $\pm$ 1.2^a^ |
| Zinc | 82.5 $\pm$1.2 | 84.2 $\pm$1.0 |
| Choline | 53.6 $\pm$1.4 | 55.9 $\pm$1.1 |
| Folate, DFE^2^ | 84.3 $\pm$1.6 | 88.6 $\pm$0.8 |
| Vitamin C | 64.1 $\pm$2.4 | 67.9 $\pm$1.5 |
| Vitamin D | 29.2 $\pm$1.4 | 33.4 $\pm$1.2 |
| Total FNI Score | 63.5 $\pm$1.2 | 67.8 $\pm$0.9 |

^1^Child food security status within the household was used to estimate food security status.

TNI/FNI scores were estimated using a simple algorithm method and National Health and Nutrition Examination Survey day 1 dietary sampling weights were applied. Mean score $\pm$SE out of a maximum score of 100.0.

^2^Folate, as DFE, Dietary Folate Equivalents

*Based on t-test, p< 0.003 to adjust for multiple comparisons using Bonferroni method. Two groups were analyzed, resulting in 1 possible pairwise comparison, calculated as 2!/(2-2)!2. With 18 nutrient scores, this led to a total of 18 (1 by 18) comparisons that were utilized in the Bonferroni adjustment of 0.05/18 for the *P* value=0.003. The shared letter means a significant difference between columns.

Supplementary Table 3. Comparison of mean usual nutrient intake among U.S. adolescents (14-17 y), by household child food security status and egg-rich diets, National Health and Nutrition Examination Survey 2007-2018^1*^

|  |  | Food Insecure | | | Food Secure | | |
| --- | --- | --- | --- | --- | --- | --- | --- |
|  |  | Non-egg consumers | Eggs as ingredients in dishes | Primarily egg dishes | Non-egg consumers | Eggs as ingredients in dishes | Primarily  egg dishes |
| Nutrient | Recommen-dation^2^  (per day) | Mean $\pm$SE  (n=264) | Mean $\pm$SE  (n=168) | Mean $\pm$SE  (n=88) | Mean $\pm$SE  (n=1504) | Mean $\pm$SE  (n=1141) | Mean $\pm$SE  (n=468) |
| Lutein+Zea, mcg | — | 886.8 $\pm$119.6^a^ | 1201.6 $\pm$191.9 | 1184.5 $\pm$1.5 | 1113.0 $\pm$70.4 | 1084.7 $\pm$50.7^b^ | 1544.1 $\pm$87.6^ab^ |
| Choline, mg^3^ | 400-550 | 217.4 $\pm$38.8^a^ | 296.4 $\pm$12.0^b^ | 364.4 $\pm$33.5 | 268.5 $\pm$10.0^c^ | 295.0 $\pm$7.9^d^ | 408.4 $\pm$17.6^abcd^ |
| Vitamin A, mcg | 485-630 | 473.7 $\pm$68.3 | 633.8 $\pm$44.2 | 707.5 $\pm$51.5 | 592.5 $\pm$20.3 | 615.0 $\pm$32.2 | 747.7 $\pm$29.1 |
| Potassium, mg^3^ | 2,300-3,000 | 2054.8 $\pm$198.0 | 2605.8$\pm$ 74.1 | 2519.0 $\pm$91.0 | 2442.7 $\pm$62.2 | 2622.8 $\pm$84.1 | 2759.1 $\pm$87.2 |
| Folate, mcg | 330 | 390.8 $\pm$70.2 | 548.0 $\pm$39.6 | 554.1 $\pm$51.7 | 504.8 $\pm$12.3 | 548.4 $\pm$14.0 | 533.1 $\pm$22.2 |
| Calcium, mg | 1100 | 768.5 $\pm$118.4 | 977.2 $\pm$52.5 | 959.5 $\pm$75.5 | 933.6 $\pm$19.1 | 1019.7 $\pm$26.5 | 1055.3 $\pm$34.7 |
| Selenium, mcg | 45 | 80.8 $\pm$14.3 | 106.6$\pm$5.1 | 122.2 $\pm$11.6 | 100.1 $\pm$3.8^a^ | 111.8 $\pm$3.3 | 128.6 $\pm$3.9^a^ |
| Magnesium, mg | 300-340 | 213.3 $\pm$35.3 | 301.6 $\pm$9.2 | 283.8 $\pm$13.5 | 270.3 $\pm$ 5.8 | 298.7 $\pm$8.5 | 306.8 $\pm$8.7 |
| Vitamin D, mcg | 10 | 3.6 $\pm$ 0.4^a^ | 4.8 $\pm$0.3 | 5.1 $\pm$0.7 | 4.6 $\pm$0.2 | 4.9 $\pm$0.2 | 6.0 $\pm$0.2^a^ |
| Iron, mg | 7.7-7.9 | 11.7 $\pm$ 1.3 | 15.6 $\pm$0.8 | 14.4 $\pm$0.7 | 13.7 $\pm$0.4 | 16.0 $\pm$0.3 | 15.8 $\pm$0.5 |
| Zinc, mg | 7.3-8.5 | 9.2 $\pm$ 1.3 | 11.0 $\pm$0.5 | 11.6 $\pm$0.8 | 10.5 $\pm$0.4 | 11.8 $\pm$0.3 | 11.8 $\pm$0.3 |
| Vitamin E, mg | 12 | 5.4 $\pm$ 0.8 | 8.3$\pm$0.4 | 8.9 $\pm$ 0.8 | 7.2 $\pm$0.2 | 8.7 $\pm$0.2 | 8.5 $\pm$0.4 |
| Vitamin B12, mcg | 2 | 4.9 $\pm$ 0.3 | 5.1 $\pm$0.2 | 5.3 $\pm$ 0.5 | 4.7 $\pm$0.2 | 5.3 $\pm$0.2 | 5.5 $\pm$ 0.2 |
| Vitamin B2, mg | 0.9-1.1 | 1.5 $\pm$0.2 | 2.1 $\pm$0.0 | 2.1 $\pm$0.1 | 1.9 $\pm$0.0^a^ | 2.1 $\pm$0.0 | 2.3 $\pm$ 0.0^a^ |
| Vitamin B1, mg | 0.9-1 | 1.3 $\pm$0.1 | 1.6 $\pm$0.0 | 1.6 $\pm$0.1 | 1.5 $\pm$0.0 | 1.7 $\pm$0.0 | 1.7 $\pm$0.0 |
| Vitamin B6, mg | 1-1.1 | 1.8 $\pm$0.1 | 2.0 $\pm$0.0 | 1.9 $\pm$ 0.1 | 1.9 $\pm$ 0.0 | 2.1 $\pm$0.0 | 2.2 $\pm$ 0.0 |
| DHA, mg | — | 30.0$\pm$0.0^a^ | 30.0$\pm$0.0^b^ | 70.0$\pm$ 0.0 | 30.0$\pm$0.0^c^ | 40.0$\pm$0.0^d^ | 70.0 $\pm$ 0.0^abcd^ |
| Vitamin C, mg | 56-63 | 61.4$\pm$8.2 | 89.8 $\pm$4.2 | 79.6 $\pm$ 6.9 | 81.3 $\pm$7.6 | 79.0 $\pm$3.7 | 92.8 $\pm$6.3 |
| Vitamin B3, mg | 11-12 | 22.5 $\pm$1.4 | 25.8 $\pm$1.1 | 24.9 $\pm$ 2.1 | 23.9 $\pm$0.6 | 26.6 $\pm$1.0 | 26.0 $\pm$0.7 |
| Protein DGA, g | 46-52 | 58.2 $\pm$ 10.9 | 80.0 $\pm$3.8 | 82.4 $\pm$ 5.8 | 72.8 $\pm$2.6^a^ | 81.8 $\pm$2.9 | 89.1 $\pm$2.3^a^ |
| Protein TFP, g | 55-75 | 58.2 $\pm$ 10.9 | 80.0 $\pm$3.8 | 82.4 $\pm$ 5.8 | 72.8 $\pm$2.6 ^a^ | 81.8 $\pm$2.9 | 89.1 $\pm$2.3^a^ |

^1^Child food security status within the household was used to estimate food security status. Lutein+Zea, Lutein + Zeaxanthin; DHA,

Docosahexaenoic acid; DGA, Dietary Guidelines for Americans; TFP, Thrifty Food Plan; — no official Dietary Reference Intakes

(DRI) (data not shown).

Estimations of Vitamin A as RAE, Retinol Activity Equivalents; Folate, as DFE, Dietary Folate Equivalents; and Vitamin E, as alpha tocopherol equivalents.

^2^Recommendation column shows the DRI: Estimated Average Requirement (EAR) or Adequate Intake (AI), and the protein recommendation according to the DGA or TFP. EAR, AI, DGA and TFP ranges for adolescents 14-18 years are dependent on sex.

^3^Estimated using the AI as an EAR is not established.

*Based on t-test, *P* < 0.0002 to adjust for multiple comparisons using Bonferroni method. Six groups were analyzed, resulting in 15 possible pairwise comparisons, calculated as 6!/(6-2)!2. With 21 nutrient markers, this led to a total of 315 (15 by 21) comparisons that were utilized in the Bonferroni adjustment of 0.05/315 for the *P* value=0.0002. The shared letter means a significant difference between columns.

Supplementary Table 4. Comparison of means of the Food Nutrient Index (FNI) and Total Nutrient Index (TNI) and component scores among U.S. adolescents (14-17 y), by household child food security status and egg-rich diets, National Health and Nutrition Examination Survey 2007-2018^1*^

|  | Food Insecure | | | Food Secure | | |
| --- | --- | --- | --- | --- | --- | --- |
| TNI Components | Non-egg consumers | Eggs as  ingredients in dishes | Primarily egg dishes | Non-egg consumers | Eggs as ingredients in dishes | Primarily  egg dishes |
|  | Mean $\pm$ SE  (n=260) | Mean $\pm$SE  (n=161) | Mean $\pm$SE  (n=94) | Mean $\pm$SE  (n=624) | Mean $\pm$SE  (n=460) | Mean $\pm$SE  (n=223) |
| Calcium | 64.8 $\pm$2.3 | 67.9 $\pm$2.2 | 68.3 $\pm$1.8 | 68.3 $\pm$1.7 | 74.8 $\pm$1.5 | 76.1 $\pm$2.3 |
| Magnesium | 54.6 $\pm$1.8^ab^ | 62.2 $\pm$1.9 | 60.3 $\pm$1.2^c^ | 61.5 $\pm$1.5^d^ | 66.9 $\pm$1.6^a^ | 71.5 $\pm$2.0^bcd^ |
| Potassium | 68.8 $\pm$2.1^ab^ | 74.4 $\pm$1.8 | 78.1 $\pm$1.5 | 73.9 $\pm$1.6 | 81.0 $\pm$1.6^a^ | 83.9 $\pm$2.0^b^ |
| Zinc | 80.6 $\pm$1.8 | 84.2 $\pm$1.6 | 87.7 $\pm$1.0 | 83.8 $\pm$1.4 | 86.8 $\pm$1.3 | 88.3 $\pm$1.8 |
| Choline | 46.4 $\pm$1.8^a^ | 53.8 $\pm$1.9^bc^ | 74.9 $\pm$1.6 ^bde^ | 50.5 $\pm$1.4^df^ | 53.4 $\pm$1.4^eg^ | 77.7 $\pm$2.2^acfg^ |
| Folate, DFE^2^ | 80.6 $\pm$2.6^a^ | 87.9 $\pm$1.5 | 92.0 $\pm$0.7 | 88.1 $\pm$1.2 | 91.9 $\pm$1.2^a^ | 91.5 $\pm$ 1.2 |
| Vitamin C | 62.0 $\pm$3.0^a^ | 71.7 $\pm$3.7 | 73.6 $\pm$2.0 | 69.6 $\pm$1.8^b^ | 72.5 $\pm$2.2 | 81.6 $\pm$2.0^ab^ |
| Vitamin D | 34.1 $\pm$2.0^a^ | 38.0 $\pm$2.6^b^ | 36.2 $\pm$1.2^c^ | 36.9 $\pm$1.8^d^ | 44.7 $\pm$2.4 | 54.2 $\pm$2.8^abcd^ |
| Total TNI Score | 61.5 $\pm$1.7^a^ | 67.5 $\pm$1.7^b^ | 71.4 $\pm$1.0 | 66.6 $\pm$1.1^c^ | 71.5 $\pm$1.3^a^ | 78.1 $\pm$1.6^bc^ |
| FNI Components | Non-egg consumers | Eggs as  ingredients in dishes | Primarily egg dishes | Non-egg consumers | Eggs as ingredients in dishes | Primarily  egg dishes |
|  | Mean $\pm$SE  (n=260) | Mean $\pm$SE  (n=161) | Mean $\pm$SE  (n=94) | Mean $\pm$SE  (n=624) | Mean $\pm$SE  (n=460) | Mean $\pm$SE  (n=223) |
| Calcium | 63.2 $\pm$2.3 | 66.8 $\pm$2.1 | 66.8 $\pm$1.6 | 67.4 $\pm$1.7 | 73.4 $\pm$1.6 | 74.9 $\pm$2.3 |
| Magnesium | 53.6 $\pm$1.8^ab^ | 61.3 $\pm$1.8 | 60.1 $\pm$1.2^c^ | 60.5 $\pm$1.5^d^ | 65.8 $\pm$1.5^a^ | 70.1 $\pm$1.8^bcd^ |
| Potassium | 68.8 $\pm$2.1^ab^ | 74.4 $\pm$1.8 | 78.1 $\pm$1.5 | 73.8 $\pm$1.6^c^ | 81.0 $\pm$1.6^a^ | 83.9 $\pm$2.0^bc^ |
| Zinc | 80.4 $\pm$1.8 | 83.4 $\pm$1.6 | 87.1 $\pm$1.0 | 82.4 $\pm$1.5 | 85.0 $\pm$1.5 | 88.0 $\pm$1.8 |
| Choline | 46.3 $\pm$1.8^ab^ | 53.8 $\pm$1.9^cd^ | 74.9 $\pm$1.6^acef^ | 50.4 $\pm$1.4^eg^ | 53.2 $\pm$1.5^fh^ | 77.6 $\pm$2.2^bdgh^ |
| Folate, DFE^2^ | 80.0 $\pm$2.6^a^ | 87.2 $\pm$1.4 | 91.5 $\pm$0.7^a^ | 86.6 $\pm$1.2 | 90.2 $\pm$1.3 | 91.0 $\pm$1.2 |
| Vitamin C | 60.1 $\pm$3.3 | 67.2 $\pm$3.7 | 70.2 $\pm$1.8 | 64.7 $\pm$1.9^a^ | 67.8 $\pm$2.3 | 77.7 $\pm$1.9^a^ |
| Vitamin D | 28.0 $\pm$2.0^a^ | 29.7 $\pm$1.9^b^ | 32.2 $\pm$1.3^c^ | 29.7 $\pm$1.6^d^ | 33.0 $\pm$1.3^e^ | 44.9 $\pm$2.2^abcde^ |
| Total FNI Score | 60.0 $\pm$1.7^abc^ | 65.5 $\pm$1.6^d^ | 70.1 $\pm$1.0^a^ | 64.4 $\pm$1.2^e^ | 68.7 $\pm$1.2^b^ | 76.0 $\pm$1.5^cde^ |

^1^Child food security status within the household was used to estimate food security status.

^2^Folate, as DFE, Dietary Folate Equivalents

*TNI/FNI scores were estimated using a simple algorithm method and National Health and Nutrition Examination Survey day 1 dietary sampling weights were applied. Mean score $\pm$SE out of a maximum score of 100.0. Based on t-test, *P* < 0.0002 to adjust for multiple comparisons using Bonferroni method. Six groups were analyzed, resulting in 15 possible pairwise comparisons, calculated as 6!/(6-2)!2. With 18 nutrient scores, this led to a total of 270 (15 by 18) comparisons that were utilized in the Bonferroni adjustment of 0.05/270 for the *P* value=0.0002. The shared letter means a significant difference between columns.

Supplementary Table 5. Comparisons of mean usual nutrient intake with the addition of one egg^1^ among U.S. adolescents (14-17 y), by household child food security status and egg-rich diets, National Health and Nutrition Examination Survey 2007-2018^2*^

|  |  | Food Insecure | | | Food Secure | | |
| --- | --- | --- | --- | --- | --- | --- | --- |
|  |  | Non-Egg  Consumers | Eggs as ingredients in Dishes | Primarily Egg Dishes | Non-Egg Consumers | Eggs as ingredients in Dishes | Primarily Egg Dishes |
| Nutrient | Recommen-dation^3^  (per day) | Mean $\pm$SE  (n=264) | Mean $\pm$SE  (n=168) | Mean $\pm$SE  (n=88) | Mean $\pm$SE  (n=1504) | Mean $\pm$SE  (n=1141) | Mean $\pm$SE  (n=468) |
| Lutein+ Zea, mcg | — | 1226.5 $\pm$74.6^a^ | 1482.6 $\pm$210.6 | 1363.1 $\pm$205.8 | 1378.2 $\pm$67.0 | 1337.4 $\pm$48.5^b^ | 1749.1 $\pm$84.7^ab^ |
| Choline, mg^4^ | 475 | 378.8 $\pm$ 24.6^a^ | 429.3 $\pm$12.2^b5^ | 496.3 $\pm$32.6 | 406.0 $\pm$9.0^c5^ | 426.1 $\pm$ 7.3^d5^ | 536.6 $\pm$17.3^abcd5^ |
| Vitamin A, mcg | 485-630 | 603.9 $\pm$ 42.7^a^ | 710.9 $\pm$41.0 | 778.5 $\pm$49.7 | 682.6 $\pm$18.5 | 701.6 $\pm$28.8 | 815.7 $\pm$27.3^a^ |
| Potassium, mg^4^ | 2,300-3,000 | 2131.6 $\pm$189.5 | 2666.2 $\pm$73.7 | 2578.1 $\pm$90.6 | 2509.1 $\pm$61.8 | 2683.6 $\pm$83.8 | 2818.8 $\pm$86.9 |
| Folate, mcg | 330 | 439.2 $\pm$54.6 | 572.4 $\pm$39.3 | 579.1 $\pm$51.7 | 531.2 $\pm$12.2 | 571.1 $\pm$13.7 | 557.7 $\pm$21.8 |
| Calcium, mg | 1100 | 809.3 $\pm$107.3 | 999.7 $\pm$52.3 | 981.8 $\pm$75.0 | 957.5 $\pm$18.9 | 1041.5 $\pm$26.3 | 1077.2 $\pm$34.4 |
| Selenium, mcg | 45 | 103.3 $\pm$ 9.4 | 122.1 $\pm$ 5.0 | 138.0 $\pm$11.2 | 116.0 $\pm$3.7^a^ | 126.2 $\pm$3.1 | 143.0 $\pm$ 3.7^a^ |
| Magnesium, mg | 300-340 | 227.8 $\pm$29.0 | 307.3 $\pm$9.2 | 289.5 $\pm$13.5 | 276.3 $\pm$5.8 | 304.0 $\pm$8.5 | 312.6 $\pm$ 8.7 |
| Vitamin D, mcg | 10 | 5.1 $\pm$0.3 | 5.9 $\pm$0.2 | 6.4 $\pm$0.6 | 5.9 $\pm$0.1^5^ | 6.0 $\pm$0.1^5^ | 6.8 $\pm$0.2 |
| Iron, mg | 7.7-7.9 | 12.7 $\pm$ 1.2 | 16.4 $\pm$0.8 | 15.2 $\pm$0.7 | 14.5 $\pm$0.4 | 16.7 $\pm$0.3 | 16.6 $\pm$0.5 |
| Zinc, mg | 7.3-8.5 | 10.1 $\pm$ 1.0 | 11.6 $\pm$0.5 | 12.2 $\pm$0.8 | 11.2 $\pm$0.4 | 12.3 $\pm$0.3 | 12.4 $\pm$0.3 |
| Vitamin E, mg | 12 | 6.3 $\pm$ 0.5^a^ | 8.8 $\pm$0.4 | 9.4 $\pm$0.9 | 7.7 $\pm$0.2 | 9.1 $\pm$0.2^a^ | 9.0 $\pm$0.4 |
| Vitamin B12, mcg | 2 | 5.3 $\pm$ 0.3 | 5.5 $\pm$0.2 | 5.6 $\pm$0.5 | 5.1 $\pm$0.2 | 5.6 $\pm$0.1 | 5.8 $\pm$ 0.2 |
| Vitamin B2, mg | 0.9-1.1 | 1.9 $\pm$0.1 | 2.2 $\pm$0.1 | 2.3 $\pm$0.1 | 2.1 $\pm$0.1^a^ | 2.3 $\pm$0.1 | 2.5 $\pm$ 0.0^a^ |
| Vitamin B1, mg | 0.9-1 | 1.3 $\pm$0.1 | 1.6 $\pm$0.0 | 1.6 $\pm$0.1 | 1.5 $\pm$0.0 | 1.7 $\pm$0.0 | 1.7 $\pm$ 0.0 |
| Vitamin B6, mg | 1-1.1 | 1.9 $\pm$ 0.1 | 2.0 $\pm$ 0.1 | 2.0 $\pm$0.1 | 1.9 $\pm$0.0 | 2.1 $\pm$0.1 | 2.2 $\pm$0.0 |
| DHA, mg | — | 30.0 $\pm$0.0^a^ | 30.0 $\pm$0.0^b^ | 70.0 $\pm$0.0 | 30.0 $\pm$0.0^c^ | 40.0 $\pm$0.0^d^ | 70.0 $\pm$0.0^abcd^ |
| Vitamin C, mg | 56-63 | 61.4 $\pm$8.2 | 89.8 $\pm$ 4.2 | 79.6 $\pm$6.9 | 81.3 $\pm$ 7.6 | 79.0 $\pm$3.7 | 92.8 $\pm$ 6.3 |
| Vitamin B3, mg | 11-12 | 22.5 $\pm$ 1.4 | 25.8 $\pm$1.1 | 24.9 $\pm$2.1 | 23.9 $\pm$0.6 | 26.6 $\pm$1.0 | 26.0 $\pm$0.7 |
| Protein DGA, g | 46-52 | 69.6 $\pm$7.2 | 86.0 $\pm$3.8 | 88.2 $\pm$5.7 | 79.0 $\pm$2.6^a^ | 87.3 $\pm$2.8 | 94.8 $\pm$ 2.3^a^ |
| Protein TFP, g | 55-75 | 69.6 $\pm$7.2 | 86.0 $\pm$3.8 | 88.2 $\pm$ 5.7 | 79.0 $\pm$2.6^a^ | 87.3 $\pm$2.8 | 94.8 $\pm$2.3^a^ |

^1^One serving size of 50 g of an “Egg, whole, boiled or poached¨ from the most current version of the Food and Nutrient Database for Dietary Studies (FNDDS) 2019-2020 (25)

^2^Lutein+Zea, Lutein + Zeaxanthin; DHA, Docosahexaenoic acid; DGA, Dietary Guidelines for Americans; TFP, Thrifty Food Plan; — no official Dietary Reference Intakes (DRI) (data not shown). Child food security status within the household was used to estimate food security status.

Estimations of Vitamin A as RAE, Retinol Activity Equivalents; Folate, as DFE, Dietary Folate Equivalents; and Vitamin E, as alpha tocopherol equivalents.

^3^Recommendation column shows the DRI: Estimated Average Requirement (EAR) or Adequate Intake (AI); or the protein recommendation according to the DGA or TFP. EAR, AI, DGA and TFP ranges for adolescents 14-18 years are dependent on sex.

^4^ Estimated using the AI as an EAR is not established.

^5^Based on t-test, p< 0.0002 to adjust for multiple comparisons using Bonferroni method. Six groups were analyzed, resulting in 15 possible pairwise comparisons, calculated as 6!/(6-2)!2. With 21 nutrient markers, this led to a total of 315 (15 by 21) comparisons that were utilized in the Bonferroni adjustment of 0.05/315 for the p value=0.0002.The shared letter means a significant difference between columns.

^*^Significant change in the usual nutrient intake with the addition of 1 egg, based on t-test, *P* <0.0004 to adjust for multiple comparisons using Bonferroni method. Six groups were analyzed by 21 nutrient markers=126 comparisons utilized in the Bonferroni adjustment of 0.05/126 for the *P* value=0.0004.

Supplementary Table 6. Comparison of means of Food Nutrient Index (FNI) and Total Nutrient Index (TNI) total and component scores among U.S. adolescents (14-17y), with the addition of an egg^1^ by household child food security status and egg-rich diets, National Health and Nutrition Examination Survey 2007-2018^2^

|  | Food Insecure | | | Food Secure | | |
| --- | --- | --- | --- | --- | --- | --- |
| TNI Components | Non-egg consumers | Eggs as ingredients in dishes | Primarily egg dishes | Non-egg consumers | Eggs as ingredients in dishes | Primarily  egg dishes |
|  | Mean $\pm$SE  (n=260) | Mean $\pm$SE  (n=161) | Mean $\pm$SE  (n=94) | Mean $\pm$SE  (n=624) | Mean $\pm$SE  (n=460) | Mean $\pm$SE  (n=223) |
| Calcium | 66.4 $\pm$ 2.3 | 69.3 $\pm$ 2.1 | 69.8 $\pm$ 1.8 | 69.7 $\pm$ 1.7 | 76.0 $\pm$ 1.5 | 77.3 $\pm$ 2.3 |
| Magnesium | 56.1 $\pm$ 1.8 | 63.6 $\pm$ 1.9 | 61.9 $\pm$ 1.2 | 62.9 $\pm$ 1.4 | 68.3 $\pm$ 1.6 | 72.8 $\pm$ 1.9 |
| Potassium | 70.9 $\pm$ 2.0 | 76.2 $\pm$ 1.8 | 80.0 $\pm$ 1.4 | 75.8 $\pm$ 1.6 | 82.7 $\pm$ 1.6 | 85.4 $\pm$ 1.9 |
| Zinc | 84.0 $\pm$ 1.6 | 87.1 $\pm$ 1.3 | 90.6 $\pm$ 0.8 | 86.9 $\pm$ 1.3 | 89.5 $\pm$ 1.2 | 91.0 $\pm$ 1.6 |
| Choline | 73.5 $\pm$ 1.4^3^ | 78.4 $\pm$ 1.4^3^ | 92.0 $\pm$ 0.7^3^ | 76.8 $\pm$ 1.1^3^ | 79.7 $\pm$ 1.1^3^ | 93.1 $\pm$ 1.0^3^ |
| Folate, DFE^4^ | 83.5 $\pm$ 2.3 | 90.4 $\pm$ 1.3 | 93.7 $\pm$ 0.6 | 90.3 $\pm$ 1.0 | 93.6 $\pm$ 1.0 | 93.5 $\pm$ 1.0 |
| Vitamin C | 62.0 $\pm$ 3.0 | 71.7 $\pm$ 3.7 | 73.6 $\pm$ 2.0 | 69.6 $\pm$ 1.8 | 72.5 $\pm$ 2.2 | 81.6 $\pm$ 2.0 |
| Vitamin D | 41.8 $\pm$ 2.0 | 45.0 $\pm$ 2.5 | 43.8 $\pm$ 1.1^3^ | 44.1 $\pm$ 1.7 | 51.5 $\pm$ 2.2 | 60.3 $\pm$ 2.6 |
| Total TNI Score | 67.3 $\pm$ 1.5 | 72.7 $\pm$ 1.6 | 75.7 $\pm$ 0.9 | 72.0 $\pm$ 1.1 | 76.7 $\pm$ 1.2 | 81.9 $\pm$ 1.4 |
| FNI Components | Non-egg consumers | Eggs as ingredients in dishes | Primarily egg dishes | Non-egg consumers | Eggs as ingredients in dishes | Primarily  egg dishes |
|  | Mean $\pm$SE  (n=260) | Mean $\pm$SE  (n=161) | Mean $\pm$SE  (n=94) | Mean $\pm$SE  (n=624) | Mean $\pm$SE  (n=460) | Mean $\pm$SE  (n=223) |
| Calcium | 64.8 $\pm$ 2.2 | 68.1 $\pm$ 2.1 | 68.3 $\pm$ 1.6 | 68.9 $\pm$ 1.7 | 74.8 $\pm$ 1.5 | 76.2 $\pm$ 2.2 |
| Magnesium | 55.1 $\pm$ 1.8 | 62.7 $\pm$ 1.8 | 61.7 $\pm$ 1.2 | 61.9 $\pm$ 1.4 | 67.3 $\pm$ 1.5 | 71.5 $\pm$ 1.8 |
| Potassium | 70.9 $\pm$ 2.0 | 76.2 $\pm$ 1.8 | 80.0 $\pm$ 1.4 | 75.7 $\pm$ 1.6 | 82.6 $\pm$ 1.6 | 85.4 $\pm$ 1.9 |
| Zinc | 83.9 $\pm$ 1.6 | 86.4 $\pm$ 1.4 | 90.2 $\pm$ 0.8 | 85.8 $\pm$ 1.3 | 88.1 $\pm$ 1.3 | 90.7 $\pm$ 1.6 |
| Choline | 73.4 $\pm$ 1.4 ^3^ | 78.3 $\pm$ 1.4 ^3^ | 92.0 $\pm$ 0.7^3^ | 76.8 $\pm$ 1.1^3^ | 79.6 $\pm$ 1.1^3^ | 93.1 $\pm$ 1.0^3^ |
| Folate, DFE^4^ | 83.2 $\pm$ 2.4 | 89.8 $\pm$ 1.2 | 93.4 $\pm$ 0.6 | 89.1 $\pm$ 1.0 | 92.4 $\pm$ 1.1 | 93.1 $\pm$ 1.0 |
| Vitamin C | 60.1 $\pm$ 3.3 | 67.2 $\pm$ 3.7 | 70.2 $\pm$ 1.8 | 64.7 $\pm$ 1.9 | 67.8 $\pm$ 2.3 | 77.7 $\pm$ 1.9 |
| Vitamin D | 35.9 $\pm$ 2.0 | 37.4 $\pm$ 1.9 | 40.0 $\pm$ 1.2^3^ | 37.4 $\pm$ 1.5 | 40.8 $\pm$ 1.3^3^ | 52.1 $\pm$ 2.1 |
| Total FNI Score | 65.9 $\pm$ 1.6 | 70.8 $\pm$ 1.5 | 74.5 $\pm$ 0.9 | 70.0 $\pm$ 1.1 | 74.2 $\pm$ 1.1 | 80.0 $\pm$ 1.3 |

^1^ One serving size of 50 g of an “Egg, whole, boiled or poached¨ from the most current version of the FNDDS 2019-2020 (25)

^2^TNI/FNI Scores were estimated using a simple algorithm method and NHANES day 1 dietary sampling weights were applied. Mean score $\pm$ SE out of a maximum score of 100.0.

^3^Significant change in the TNI and FNI with the addition of one egg, based on t-test, *P* <0.0005 to adjust for multiple comparisons using Bonferroni method. Six groups were analyzed by 18 nutrient scores=108 comparisons utilized in the Bonferroni adjustment of 0.05/108 for the *P* value=0.0005.

^4^Folate, as DFE, Dietary Folate Equivalents
